# Quality of Life and Medication Adherence in Patients With Major Depressive Disorder: An Interim Analysis of a Randomized Study

**DOI:** 10.7759/cureus.39997

**Published:** 2023-06-05

**Authors:** N Simple Santi, Sashi B Biswal, Birendra Narayan Naik, Jyoti Prakash Sahoo, Bhabagrahi Rath

**Affiliations:** 1 Pharmacology, Veer Surendra Sai Institute of Medical Sciences and Research (VIMSAR), Burla, IND; 2 Psychiatry, Veer Surendra Sai Institute of Medical Sciences and Research (VIMSAR), Burla, IND; 3 Pharmacology, Kalinga Institute of Medical Science, Bhubaneswar, IND

**Keywords:** serotonin receptor, serotonin, escitalopram, vortioxetine, vilazodone, morisky medication adherence scale-8, short form 36, antidepressant drug, major depressive disorder, hamilton depression rating scale (ham-d)

## Abstract

Background and objectives: Quality of life and medication adherence worsen in untreated depressed individuals. Studies examining how vilazodone, escitalopram, and vortioxetine affect these factors are few and far between. Our study’s objectives were to determine the change in SF-36 at 12 weeks and the association between treatment outcome and medication adherence.

Methods: This is an interim analysis of a randomized, open-label, three-arm ongoing study. The participants were evaluated at baseline, four, eight, and 12 weeks after being randomly assigned to take either vilazodone (20-40 mg/d), escitalopram (10-20 mg/d), or vortioxetine (5-20 mg/d). This study is registered with CTRI, 2022/07/043808.

Results: Of 71 recruited participants, 49 (69%) completed the 12-week visit. The median scores of physical components of SF-36 for the three groups were 35.5, 35.0, and 35.0 at baseline (p=0.76) and 51.0, 49.5, and 53.0 (p<0.001) at 12 weeks respectively. Their corresponding median SF-36 scores for mental components were 43.0, 43.0, and 44.0 at baseline (p=0.34) and 66.0, 63.5, and 70.0 (p<0.001) at 12 weeks. The post hoc analysis yielded a significant difference (p<0.001) regarding SF-36 scores. MMAS-8 scores among the participants were similar (p=0.22) at 12 weeks. Higher medication adherence was associated with lesser depressive symptoms (r= -0.46, p=0.001).

Conclusion: As per this interim analysis, vortioxetine substantially impacted the SF-36 scores, juxtaposed with vilazodone and escitalopram. The participants’ clinical improvements were reflected by their adherence levels. These effects need to be probed further.

## Introduction

Major depressive disorder (MDD) has been acknowledged as an essential public health issue due to its detrimental effects on individuals, their families, and society [1]. Worldwide, the prevalence of depressive disorder has surged at a previously unheard-of rate during the past 20 years [2]. Conforming to the most recent figures, India faces an annual prevalence of 15.9% regarding MDD [3]. The depressive patient's quality of life and adherence shrivel noticeably with time, worsening the disease. Adherence to antidepressant medications is currently deemed the decisive horizon in managing MDD [4-6]. The five facets of health-related quality of life (HRQoL) embody physical, psychological, social, overall life satisfaction and well-being, and perceptions of health status [7].

Although there are a handful of effective antidepressants available, it is unexplored what drug improves the quality of life the most. Moreover, not much is known regarding how drug adherence could lower depressive symptoms [8]. We went with three antidepressants, namely, escitalopram, a selective serotonin receptor inhibitor (SSRI); vilazodone, SSRI with additional partial agonistic activity at 5-HT1A receptors; and vortioxetine, a serotonin modulator as well as transporter inhibitor for our study based on literature review [9-11]. This study was underpinned by the contention that novel drugs exhibiting promising antidepressant effects could aid in bettering the HRQoL of patients with MDD. Recent studies [12-15] advocate the betterment of HRQoL and medication adherence in such patients on regular antidepressants.

This study aimed to determine the quality of life and medication adherence in patients with MDD after 12-week antidepressant monotherapy of vilazodone, escitalopram, or vortioxetine. Here we report the interim results focused on changes in the physical and mental component of the Short Form-36 items version (SF-36) [16] and Morisky's Medication Adherence Scale (MMAS-8) [17] scores of a larger ongoing study.

## Materials and methods

Patients with MDD are being assessed for the effects of three drugs, namely vilazodone, escitalopram, and vortioxetine, on their quality of life and medication adherence in this three-arm, parallel-group, randomized, open-label study. Enrolment of participants began in July 2022 at the Department of Psychiatry, VIMSAR, Burla, India. The participants or their relatives gave written informed consent before group allocation. The Institutional Ethics Committee, Veer Surendra Sai Institute of Medical Sciences and Research (VIMSAR), Burla, India, granted us ethical approval (No. 029-2022/I-S-T/03 dated 17.05.2022) prior to study initiation. We registered this study with the Clinical Trial Registry, India (CTRI/2022/07/043808). The study complied with institutional statutes, the Declaration of Helsinki, and the International Council for Harmonization's standards for Good Clinical Practice.

Study participants

Women and men aged 18-65 years diagnosed with MDD at psychiatry outpatient department (OPD) of VIMSAR with a score of ≥ 24 on the HDRS-17 items version [18], were included. Patients who have an organic brain disease or psychotic symptoms, renal impairment (estimated glomerular filtration rate 45mL/min/1.73m2), cardiovascular events within the last six months, alanine transaminase (ALT) or aspartate transaminase (AST) levels above 150% the upper limit of normal, serum triglyceride level greater than 400 mg/dL and pregnant women and nursing mothers were excluded from this study. The participants had the power to withdraw their consent without any justification.

Study design and endpoints

The eligible participants were randomly assigned in a 1:1:1 ratio to embark on treatment with either vilazodone tablets (20-40 mg once daily; group A), escitalopram tablets (10-20 mg once daily; group B), or vortioxetine tablets (5-20 mg once daily; group C). We achieved this by employing permuted block randomization with block sizes of 12 and 24. Additionally, we stratified the randomization by depressive disorder (treatment naïve or on antidepressant medications for < 6 months) and gender (female or male).

The change in the physical and mental components of the Short Form-36 items version (SF-36) score from baseline at week 12 served as the primary objective for this interim analysis. The secondary objectives included the change in Morisky’s Medication Adherence Scale-8 items version (MMAS-8) score from baseline at week 12 and the impact of adherence level on treatment outcome (tracked through a change in HDRS score). We centered these assessments on the per-protocol (PP) population.

Study procedure

All participants got either of the following antidepressants throughout the study: vilazodone (20-40 mg tablet, once daily orally; group A), escitalopram (10-20 mg tablet, once daily orally; group B), or vortioxetine (5-20 mg tablet, once daily orally; group C). Participants received these medications free of charge from the principal investigator. The psychiatrist tailored their doses based on the participant's clinical response to the recommended drugs. The study drugs were never crossed over. Each patient encountered a comprehensive evaluation of their physical and mental health at the baseline visit. We slated subsequent visits for the participants at four, eight, and 12 weeks.

All participants were assessed for change in their quality of life with the Short Form-36 items version (SF-36) [16]. This questionnaire includes distinct elements for evaluating the physical and mental components of one's quality of life. Higher scores indicate better quality of life. The participants' medication adherence was measured through Morisky's Medication Adherence Scale-8 items version (MMAS-8) [17]. The overall score ranges from 0 to 8, with values <6, 6-7, and 8 denoting low, moderate, and high medication adherence, respectively. Moreover, we correlated the degree of adherence at 12 weeks with the treatment outcomes assessed by the change in the Hamilton Depression Rating Scale-17 items version (HDRS) [18] scores. Lower HDRS scores imply better treatment outcomes and reduced depressive symptoms.

Statistical analysis

The sample size was set for the overall study’s primary endpoint. With a mean difference in HDRS of 10.0 from baseline and a standard deviation of 2.0, 87 patients (29 in each group) would be necessitated to determine a change in HDRS with an 80% power at a 0.05 two-sided significance level. Considering a 10% dropout or loss to follow-up, 96 patients (32 in each group) were finalized for this study. We mapped an interim analysis following the first 48 participants’ 12-week visit.

The Shapiro-Wilk test was applied to ascertain the data’s normality. The continuous and categorical variables were expressed as mean with SD or median with IQR and frequency with proportion, respectively. The sociodemographic variables were compared using Pearson's chi-square or Fisher's exact tests. The Kruskal-Wallis test was used to compare the physical and mental component scores of SF-36. The post hoc analyses were done with the Bonferroni test. We used R software v. 4.2.2 (RStudio: Integrated Development for R. RStudio, PBC, Boston, MA) [19] for data analysis. All statistical tests were two-tailed, and a p-value < 0.05 was considered statistically significant.

## Results

**Figure 1:**
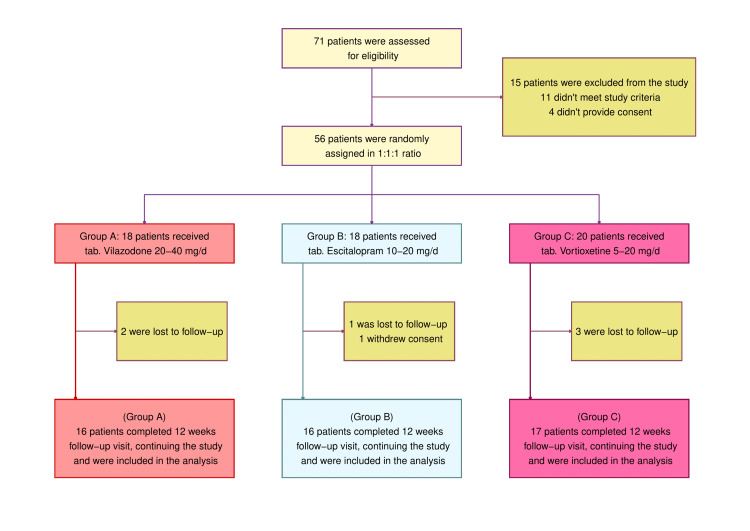
CONSORT diagram CONSORT (consolidated standards of reporting trials) diagram shows the screening, randomization, and completion of study visits till the interim analysis. Eleven patients were ineligible according to the study criteria. Ten did not come within 18-65 years of age, and one was 12 weeks pregnant at the baseline visit. This interim analysis was done in the per-protocol (PP) population (n = 49).

For this study, 71 patients were screened for eligibility; 10 who did not meet the age criteria, one pregnant woman, and four who denied consent were excluded from the study. The remaining 56 patients were randomized into one of three study groups. Six were lost to follow-up (two from the vilazodone arm, one from the escitalopram arm, and three from the vortioxetine arm) and one from the escitalopram arm withdrew her consent. Forty-nine participants were analyzed in this interim analysis (24 females and 25 males; 16 each in the vilazodone and escitalopram arms and 17 in the vortioxetine arm) (Figure 1). Participants in the three study groups had similar baseline characteristics (Table 1).

**Table 1:** Baseline characteristics of the study population (n = 49) The continuous variables were expressed as either mean ± standard deviation or median (inter-quartile range). The categorical values were presented as n (%). BMI: Body mass index; HDRS: Hamilton Depression Rating Scale-17 items version; SF-36: Short Form-36.

Parameters	Total (n = 49)	Group A Vilazodone (n = 16)	Group B Escitalopram (n = 16)	Group C Vortioxetine (n = 17)	p-Value
Age (years)	43.9 ± 12.2	45.8 ± 11.2	41.4 ± 11.9	44.4 ± 13.7	0.618
Age group					
≤ 50 years	32 (65.3%)	11 (68.8%)	11 (68.8%)	10 (58.8%)	0.789
>50 years	17 (34.7%)	5 (31.2%)	5 (31.2%)	7 (41.2%)	
Gender					
Female	25 (51.0%)	8 (50.0%)	8 (50.0%)	9 (52.9%)	0.981
Male	24 (49.0%)	8 (50.0%)	8 (50.0%)	8 (47.1%)	
Duration of disease					
Treatment naïve	25 (51.0%)	8 (50.0%)	8 (50.0%)	9 (52.9%)	0.981
Duration < 6 months	24 (49.0%)	8 (50.0%)	8 (50.0%)	8 (47.1%)	
BMI (kg/m^2^)	27.3 ± 4.8	27.4 ± 4.1	27.6 ± 5.2	27.1 ± 3.9	0.128
HDRS	30.0 (29.0 - 31.0)	30.0 (29.0 - 31.0)	29.5 (29.0 - 30.0)	29.0 (29.0 - 32.0)	0.763
SF-36 (Physical component)	35.0 (34.5 - 36.0)	35.5 (34.8 - 36.0)	35.0 (34.0 - 36.3)	35.0 (35.0 - 36.0)	0.764
SF-36 (Mental component)	43.5 (42.5 - 44.2)	43.0 (42.0 - 44.2)	43.0 (42.0 - 44.2)	44.0 (43.0 - 44.0)	0.341

**Figure 2:**
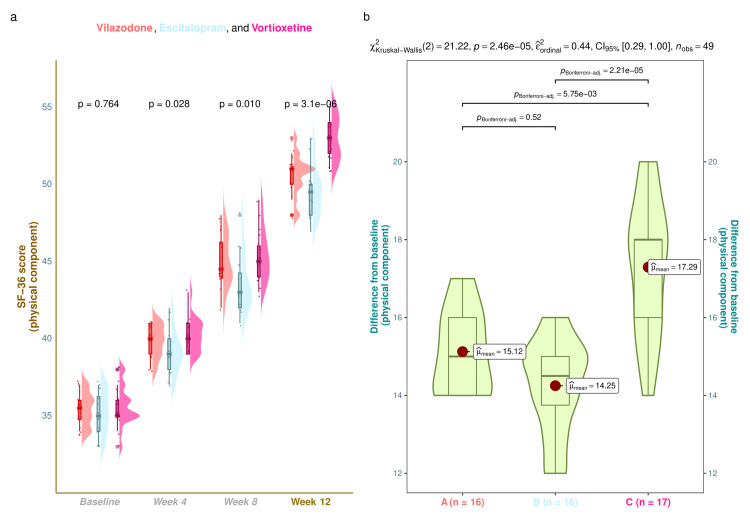
SF-36 (physical component) scores of the study participants These figures illustrate the physical component scores of SF-36 of all three groups' participants. Part a shows the raincloud plots (a combination of half-eye, box-whisker, and jitter plots) demonstrating the physical component scores of SF-36 at the baseline and follow-up visits. The inter-group comparisons at each visit were made with the Kruskal-Wallis test. Part b shows the post hoc analysis of differences in scores from the baseline. The Bonferroni test was applied for the same.

**Figure 3:**
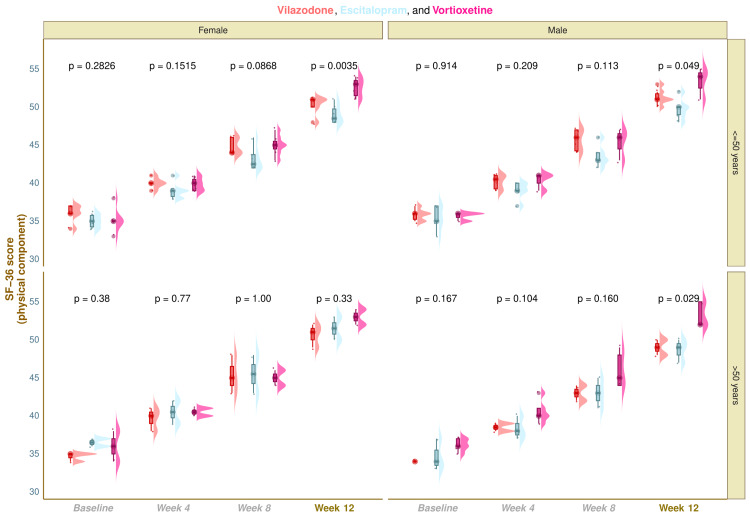
Subgroup analysis of SF-36 (physical component) scores These figures show subgroup analysis of the physical component scores of SF-36 of all three groups' participants as raincloud plots. The horizontal and vertical grids segregate the study participants on gender (female and male) and age (≤ 50 years and > 50 years) basis, respectively. The inter-group comparisons at each visit were performed with the Kruskal-Wallis test.

The physical component scores of SF-36 of the study population are shown in Figure 2. The median SF-36 (physical component) scores were 35.5 (34.8-36.0) at baseline, 40.0 (39.0-41.0) at four weeks, 44.5 (44.0-46.3) at eight weeks, and 51.0 (50.0-51.0) at 12 weeks in vilazodone group (mean difference from baseline: 15.1 (95% confidence interval (CI): 14.5-15.7); p < 0.001), 35.0 (34.0-36.3) at baseline, 39.0 (38.0-40.0) at four weeks, 43.0 (42.0-44.3) at eight weeks, and 49.5 (48.0-50.0) at 12 weeks in the escitalopram group (mean difference from baseline: 14.3 (95% CI: 13.6-14.9); p < 0.001), and 35.0 (35.0-36.0) at baseline, 40.0 (39.0-41.0) at four weeks, 45.0 (44.0-46.0) at eight weeks, and 53.0 (52.0-54.0) at 12 weeks in vortioxetine group (mean difference from baseline: 17.3 (95% CI: 16.4-18.2); p < 0.001), respectively (Figure 2a). Following 12-week antidepressant monotherapy, the intragroup comparison yielded that the physical component scores of SF-36 for all three study groups had notably burgeoned (p < 0.001). Furthermore, intergroup comparisons revealed statistically significant differences (p < 0.001). We applied the Bonferroni test while performing the post hoc analysis. It illuminated that the vortioxetine group had statistically significant differences (p < 0.001) weighed against the other two study groups (Figure 2b). Subgroup analysis of SF-36 (physical component) scores revealed that younger females and elderly males in the vortioxetine group had statistically significant improvement in the quality-of-life scores as compared to other participants (Figure 3).

**Figure 4:**
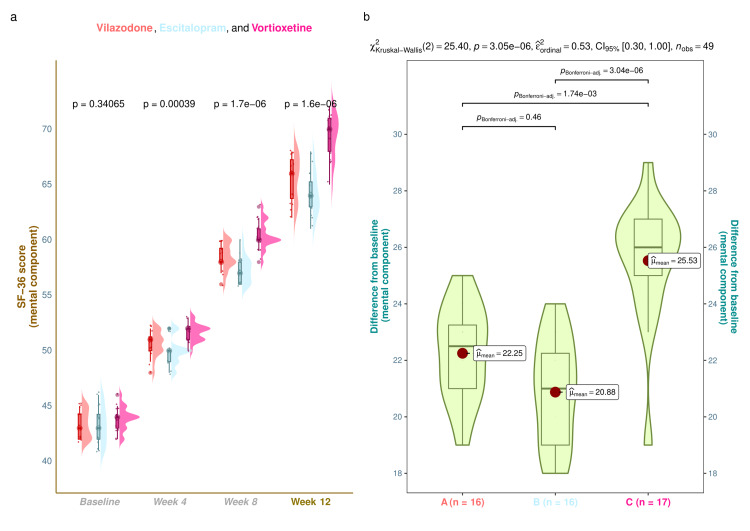
SF-36 (mental component) scores of the study participants These figures illustrate the mental component scores of SF-36 of all three groups' participants. Part a shows the raincloud plots (a combination of half-eye, box-whisker, and jitter plots) demonstrating the mental component scores of SF-36 at the baseline and follow-up visits. The inter-group comparisons at each visit were made with the Kruskal-Wallis test. Part b shows the post hoc analysis of differences in scores from the baseline. The Bonferroni test was applied for the same.

**Figure 5:**
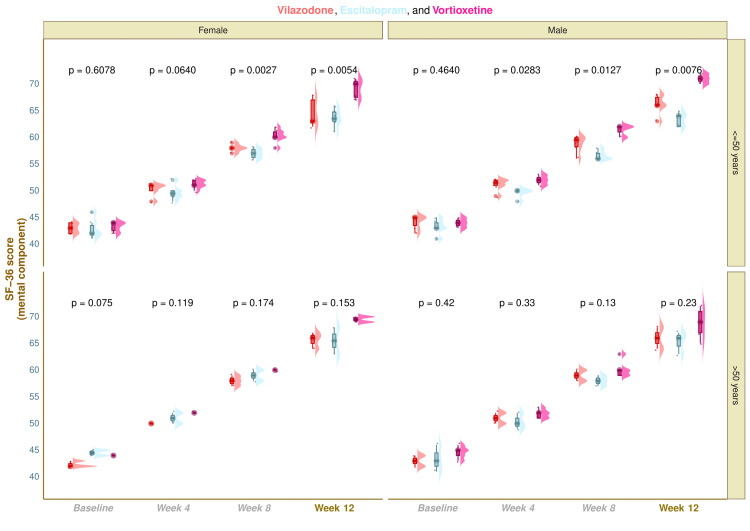
Subgroup analysis of SF-36 (mental component) scores These figures show subgroup analysis of the mental component scores of SF-36 of all 3 groups' participants as raincloud plots. The horizontal and vertical grids segregate the study participants on gender (female and male) and age (≤ 50 years and > 50 years) basis, respectively. The inter-group comparisons at each visit were performed with the Kruskal-Wallis test.

The mental component scores of SF-36 of the study population are shown in Figure 4. The median SF-36 (mental component) scores were 43.0 (42.0-44.2) at baseline, 51.0 (50.0-51.3) at four weeks, 58.0 (58.0-59.3) at eight weeks, and 66.0 (63.8-67.3) at 12 weeks in vilazodone group (mean difference from baseline: 22.3 (95% CI: 21.4-23.1); p < 0.001), 43.0 (42.0-44.2) at baseline, 50.0 (49.0-50.0) at four weeks, 57.0 (56.0-58.0) at eight weeks, and 64.0 (63.0-65.3) at 12 weeks in escitalopram group (mean difference from baseline: 20.9 (95% CI: 19.9-21.9); p < 0.001), and 44.0 (43.0-44.0) at baseline, 52.0 (51.0-52.0) at four weeks, 60.0 (60.0-61.0) at eight weeks, and 70.0 (68.0-71.0) at 12 weeks in vortioxetine group (mean difference from baseline: 25.5 (95% CI: 24.4-26.7); p < 0.001), respectively (Figure 4a). Following 12-week antidepressant monotherapy, the intragroup comparison yielded that the mental component scores of SF-36 for all three study groups had remarkably mounted (p < 0.001). Furthermore, intergroup comparisons revealed statistically significant differences (p < 0.001). We applied the Bonferroni test while performing the post hoc analysis. It foregrounded that the vortioxetine group had statistically significant differences (p < 0.001) weighed against the other two study groups (Figure 4b). Subgroup analysis of SF-36 (mental component) scores unveiled that the younger females and males in the vortioxetine group had statistically significant improvement in the quality-of-life scores as compared to elderly individuals and other study groups (Figure 5).

Medication adherence and treatment outcomes, i.e., antidepressant effects of the drugs, were evaluated with Morisky's Medication Adherence Scale-8 items version (MMAS-8) and the Hamilton Depression Rating Scale-17 items version (HDRS), respectively (Table 2). All three groups' participants had reduced HDRS scores after 12 weeks of intervention. The inter-group differences in HDRS scores from baseline were clinically and statistically significant (p = 0.021). Moderate to high-level medication adherence was observed among most of the study participants.

**Table 2:** Adherence and treatment outcome in the study population (n = 49) The treatment outcome and medication adherence were evaluated with HDRS and MMAS-8 scales. The p-values were calculated using the chi-square (χ2) and Kruskal-Wallis tests for the categorical and continuous data, respectively. HDRS: Hamilton Depression Rating Scale-17 items version; MMAS-8: Morisky's Medication Adherence Scale-8 items version.

Parameters	Group A Vilazodone (n=16)	Group B Escitalopram (n=16)	Group C Vortioxetine (n=17)	p-Value
Treatment outcome (assessed with HDRS)				
HDRS score at baseline	30.0 (29.0 - 31.0)	29.5 (29.0 - 30.0)	29.0 (29.0 - 32.0)	0.763
HDRS score at 4 weeks	27.0 (26.0 - 28.3)	27.0 (26.0 - 28.0)	26.0 (25.0 - 28.0)	0.671
HDRS score at 8 weeks	24.0 (23.0 - 25.0)	23.0 (23.0 - 24.0)	22.0 (22.0 - 23.0)	0.094
HDRS score at 12 weeks	19.5 (18.0 - 21.0)	19.5 (18.8 - 20.0)	18.0 (18.0 - 20.0)	0.182
Difference in HDRS from baseline	-10.0 (-11.0 to -9.0)	-10.0 (-11.0 to -9.0)	-11.0 (-12.0 to -11.0)	0.021
Difference in HDRS > 10 from baseline, n (%)	6 (37.5%)	7 (43.8%)	13 (76.5%)	0.003
Medication adherence (assessed with MMAS-8)				
MMAS-8 score at 12 weeks	6.0 (6.0 - 7.0)	6.0 (6.0 - 7.0)	7.0 (6.0 - 7.0)	0.223
High adherence (score = 8), n (%)	1 (6.2%)	2 (12.5%)	4 (23.5%)	0.355
Moderate adherence (score = 6-7), n (%)	12 (75.0%)	12 (75.0%)	10 (58.8%)	0.505
Low adherence (score < 6), n (%)	3 (18.8%)	2 (12.5%)	3 (17.7%)	0.877

**Figure 6:**
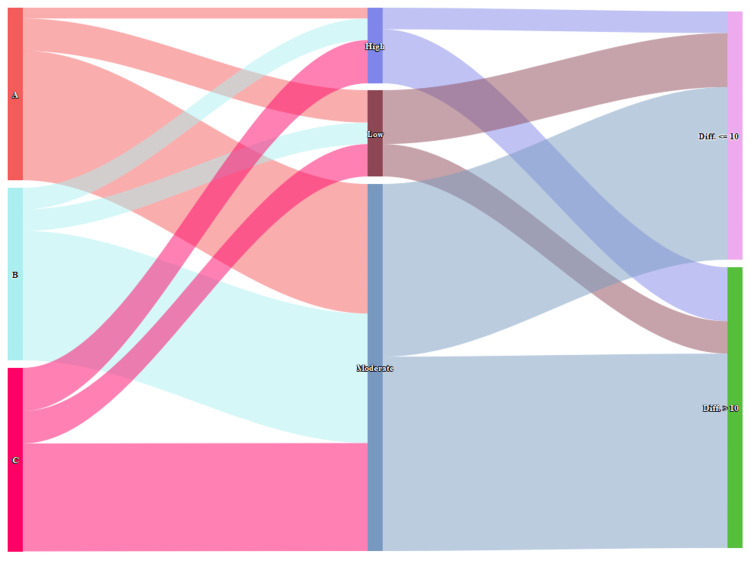
Association between medication adherence level and treatment outcomes in the three study groups The Sankey diagram shows participants of the three groups in the 1st column, medication adherence levels in the 2nd column, and the reduction in HDRS scores in the 3rd column. The width of the connecting band between any two parameters indicates their degree of association.

**Figure 7:**
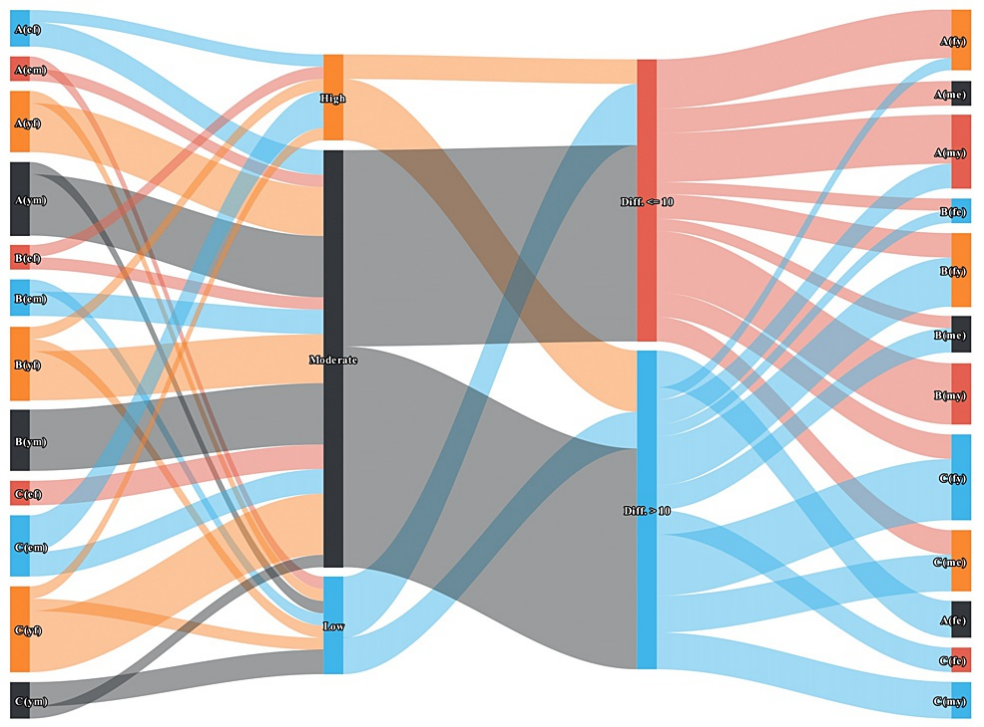
Subgroup analysis of the association between medication adherence level and treatment outcome The Sankey diagram shows subgroup analysis (based on gender and age) of association between medication adherence and treatment outcomes. The 1st and 4th columns represent younger and elderly female and male participants of the three study groups. The 2nd and 3rd columns represent the medication adherence levels and the reduction in HDRS scores, respectively. The width of the connecting band between any two parameters indicates their degree of association. A, B, and C are the study groups. Letters in parentheses indicate as follows: ef or fe – elderly females, em or me – elderly males, yf or fy – younger females, ym or my – younger males.

The Sankey diagram in Figure 6 illustrates the association between the degree of medication adherence and the difference in HDRS scores from baseline in the participants. The three columns represent the three study groups (“A”, “B”, and “C”), medication adherence (high, low, and moderate), and reduction in HDRS score (< = 10 and > 10), respectively. Higher medication adherence was associated with lesser depressive symptoms (r= -0.46, 95% CI: -0.66 to -0.20, p=0.001). We also did subgroup analysis for the association on the basis of gender and age (Figure 7). It depicted that participants in the vortioxetine group (regardless of gender and age) had pronounced reductions in HDRS scores, which were reflected by their adherence levels. Therefore, better treatment outcomes could be anticipated among the participants with high medication adherence.

## Discussion

According to this interim analysis of an ongoing study, vortioxetine caused clinically and statistically significant improvement in physical and mental component scores of SF-36 at 12 weeks compared to escitalopram and vilazodone. After 12 weeks of intervention, the HDRS scores of most study participants, who generally stayed highly compliant with antidepressant medication, had improved. Medication adherence and depressive symptoms had an explicit negative correlation. Our results regarding the SF-36 and MMAS-8 scores matched those from two other studies [12,15].

The daily doses for the two intervention arm participants were 20-40 mg of vilazodone and 5-20 mg of vortioxetine, whereas the daily dose for the control arm participants was 10-20 mg of escitalopram. The control drug, i.e., escitalopram, an SSRI, has only one mode of action, but vilazodone has the additional perk of being a partial agonist at the 5-HT1A receptor. However, vortioxetine modulates serotonin receptors and impedes serotonin transport. With these pluses, we discovered that the vortioxetine group's corresponding drops in HDRS and MADRS scores were more pronounced than in the other two groups [20]. In light of these contrasting data, vortioxetine monotherapy might be considered as a prospective treatment option for MDD.

Irrespective of their assigned groups, all participants received free medication throughout the trial. This might have incentivized a low attrition rate, which contributed to the high adherence of study participants reflected by higher MMAS-8 scores and a consistent increase in SF-36 scores over time. Vortioxetine has the potential to emerge as a first-line antidepressant in the coming years, focusing on its eclectic impact on quality of life, adherence level, efficacy, and safety parameters [13-15]. According to our prior investigations [20], high attrition rates, frequent visits, better quality of life, and high drug compliance are crucial for effective antidepressant activity. This study examines each preceding variable, enabling it to illuminate what transpired. However, since this was simply a preliminary analysis of a larger study, these findings must be considered tentative. Further evaluations are necessary to assess whether the quality of life and adherence remain after a longer follow-up.

This study's key strengths were permuted block randomization and assessment of HRQoL and medication adherence with SF-36 [16] and MMAS-8 [17]. The added advantages were the higher follow-up visits and evaluation of depressive symptoms with widely-accepted and routinely-used HDRS [18]. There are a few limitations to our study as well. First, the open-label trial design might be blamed for dropouts and potential reporting biases regarding the quality of life. It affected the study's internal validity. Second, no fee was received for the antidepressants used in this study. The study drugs' affordability might restrict the study findings' practicality. Third, these findings depend on an interim analysis of a more extensive, ongoing study. The consistency between these findings and those from the ongoing larger study has to be more clearly established.

## Conclusions

As per this interim analysis, participants in the vortioxetine group had a significant improvement in their quality of life after 12 weeks of intervention, contrasted with the vilazodone and escitalopram groups. The participants' clinical improvements were reflected in their medication adherence levels. These effects need to be investigated further.
